# To Arrest Acute Endocarditis

**Published:** 1901-10-19

**Authors:** 


### TO ARREST ACUTE ENDOCARDITIS.

Dr. Caton, of Liverpool, has devised a method of treating acute rheumatism in which he has complete confidence, even after an experience of 500 cases. Undoubtedly Dr. Caton is right in considering the prevention of endocardial change as the great thing to aim at in the treatment of this disease. So far as the rheumatism is concerned the plan he adopts is the use of salicylates, with occasional chologogues and a light diet, the patient being kept strictly in bed for several weeks, the whole body except the head being clothed in flannel. Any lingering pains are to be dissipated by the use of small blisters locally. Under this treatment the proportion of cases in which valvulitis occurs is comparatively small. If, however, there should be the slightest threatening of valvulitis, Dr. Caton adopts what he describes as a triple method of treatment—bed, blisters, and sodium iodide. For six weeks the patient must lie in bed with the head low. He must not leave his bed nor raise himself for any purpose; sleep is encouraged, pain prevented, and all mental excitement avoided; the diet is to be light and unstimulating. A succession of small blisters—each about the size of a florin—are to be applied to the wall of the chest, between the clavicle and the nipple. Only one is to be used at a time, and each is to be followed by a small poultice. Lastly, sodium iodide is to be administered internally. The results of this triple method Dr. Caton considers to be vastly superior to those obtained if the rheumatism be treated solely and the heart be left to itself, as formely was the custom. It is to be noted that Dr. Caton admits that this treatment appears to be of no use unless it be commenced early—say within the first two or three weeks.

